# *Cuminum cyminum* L. Essential Oil: A Promising Antibacterial and Antivirulence Agent Against Multidrug-Resistant *Staphylococcus aureus*

**DOI:** 10.3389/fmicb.2021.667833

**Published:** 2021-08-04

**Authors:** Aram Sharifi, Abdolmajid Mohammadzadeh, Taghi Zahraei Salehi, Pezhman Mahmoodi, Alireza Nourian

**Affiliations:** ^1^Department of Pathobiology, Faculty of Veterinary Science, Bu-Ali Sina University, Hamedan, Iran; ^2^Department of Microbiology, Faculty of Veterinary Medicine, University of Tehran, Tehran, Iran

**Keywords:** antivirulence factor, *Cuminum cyminum* L., *Staphylococcus aureus*, quorum sensing, NorA efflux pump

## Abstract

*Cuminum cyminum* L. (cumin) is valued for its aromatic and medicinal properties. There are several reports of antibacterial activity of *C. cyminum* essential oil (CcEO). Accordingly, the present study was conducted to investigate the mechanism(s) of action of the CcEO against multidrug-resistant (MDR) *Staphylococcus aureus*. Therefore, 10 *S*. *aureus* MDR isolates, obtained from different sources, were selected based on the antibiotic susceptibility patterns and the Clinical and Laboratory Standards Institute definition and subjected to the examinations. Our results exhibited promising bacteriostatic and bactericidal properties of the CcEO. The minimum inhibitory concentration (MIC) and the minimum bactericidal concentration values ranged from 5 to 10 and 10 to 20 μL ⋅ mL^–1^, respectively. Scanning electron microscope was used to assess the bacterial cell structure and morphology after the induction with 1/2 MIC concentration of the CcEO. The observed morphological changes appeared to be deformation of the cell membrane and destruction of the cells. In the case of quorum sensing inhibitory potential, treatment of *S. aureus* isolates with the sub-MIC concentrations (1/2 MIC) of the CcEO significantly reduced the *hld* expression (3.13-fold downregulation), which considerably controls *S. aureus* quorum-sensing accessory regulator system. Another virulence factor influenced by the CcEO was the polysaccharide intercellular adhesion production system, as an important component of cell–cell adhesion and biofilm formation. Consequently, the expression level of the intercellular adhesion (*ica*) locus in the *S. aureus* cells was examined following treatment with CcEO. The results showed significant decrease (−3.3-fold) in *ica* expression, indicating that the CcEO could potentially interfere with the process of biofilm formation. Using the ethidium bromide efflux inhibition assay, the *S. aureus* NorA efflux pump was phenotypically but not genotypically (in quantitative polymerase chain reaction assay) affected by the CcEO treatment. Using gas chromatography–mass spectrometry analysis, cuminic aldehyde (38.26%), α,β-dihydroxyethylbenzene (29.16%), 2-caren-10-al (11.20%), and γ-terpinene (6.49%) were the most detected compounds. The antibacterial and antivirulence action of the CcEO at sub-MIC concentrations means that no microbial resistance will be promoted and developed after the treatment with this agent. These findings revealed that the CcEO is a promising antibacterial agent to control infections caused by the MDR *S. aureus* strains.

## Introduction

Infectious diseases are known as the second leading cause of death in the world. One of the most important causes of infectious diseases is *Staphylococcus aureus*, which has the ability to cause various diseases from minor skin and soft tissue infections to life-threatening diseases such as pneumonia, severe sepsis, and fatal infections (Bezerra Filho et al., 2020; Guo et al., 2020). Today, the resistance rates of *S. aureus* infection and emergence of multidrug-resistant (MDR) strains are increasing, making the *S. aureus* infections treatment more difficult. Accordingly, the emergence and the spread of MDR *S. aureus* bacteria are a global threat for the therapeutic management of staphylococcal infections (Guo et al., 2020; Xu et al., 2020). Thus, it is necessary to identify new drugs that can serve as an alternative treatment of infections caused by MDR *S. aureus*. One appropriate approach in this area is the study of local medicinal plants with ability for antimicrobial and antivirulence properties (Sharifi et al., 2018a). Plant essential oils (EOs) are aromatic oily liquids that are well known for their different biological activities such as antibacterial activities. They contain various bioactive compounds with destructive effects on different parts of pathogen and MDR bacteria. This property makes them attractive to be used for therapeutic and food preservative purposes (Fridman, 2006; Tongnuanchan and Benjakul, 2014).

In the case of antivirulence potentials, some plant EOs interfere in microbial cell–cell communication processes, commonly called quorum sensing (QS) (Kerekes et al., 2013; Sharifi et al., 2018a). QS system, which operates by autoinducer (AI) molecules, modifies gene expression in response to the population density (Hammer and Bassler, 2003). This system is associated with the bacterial biofilm formation and antibiotic resistance, as well as bacterial proliferation. Therefore, QS inhibition is considered as a good strategy to control pathogens especially MDR bacteria (Hammer and Bassler, 2003). Previous studies have checked the expression level of QS-related genes as an indicator for quorum sensing inhibitory (QSI) potential of different agents (Kim et al., 2016; Sharifi et al., 2018b). In *S. aureus*, RNAIII transcript produced by *hld* gene is considered as the most effector molecule in staphylococcal QS system (Yarwood and Schlievert, 2003; Sharifi et al., 2018a). Hence, previous studies have assessed the level of *hld* expression to determine the anti-QS activity of agents against *S. aureus* (Sharifi et al., 2018a).

Another important factor in the development of resistance in bacteria is the formation of biofilms. In *S. aureus*, biofilm started to form by the production of polysaccharide intercellular adhesion (PIA). PIA is produced by enzymes encoded by the intercellular adhesion (*ica*) locus, which comprises four intercellular adhesion genes: *icaA*, *icaB*, *icaC*, and *icaD*. The expression of the *ica* operon and biofilm formation are tightly regulated by *icaR* under *in vitro* conditions (Cramton et al., 1999; Nuryastuti et al., 2009).

Previous researches have demonstrated that microorganisms within biofilms are more resistant to antimicrobial treatment (up to 1,000-fold) than their planktonic forms (Nuryastuti et al., 2009). Accordingly, current researches have been focused on identifying new compounds, which are potent against both planktonic and biofilm forms of growth (Nuryastuti et al., 2009; Sharifi et al., 2018a).

Another mechanism for creating and increasing microbial resistance is the activity of efflux pumps. Efflux pumps are transmembrane proteins with the capacity to expel or exchange toxic compounds such as antibiotics from bacterial cells, thus allowing bacterial survival (Costa et al., 2016; Espinoza et al., 2019). Studies show that in *S. aureus*, the NorA pump (encoded by *norA* gene) is one of the most important and active pumps that are overexpressed in 43% of *S. aureus* strains. NorA is responsible for the efflux of various compounds, such as hydrophilic fluoroquinolone antibiotics; dyes, such as acridine; ethidium bromide (EtBr); and some antimicrobial agents (Costa et al., 2016; Espinoza et al., 2019).

Today, many studies are being done to find alternative drugs to antibiotics; one of the candidates in this field are medicinal plants and their derivatives such as plant EOs and plant extracts. Plant EOs have been used in food preservation, pharmaceutical therapies, alternative medicine, and natural therapies (Seow et al., 2014; Sharifi et al., 2018a).

*Cuminum cyminum* L., known as cumin or Jeera (in Persian zeera) belonging to the *Apiaceae* family, is an aromatic herb, spice, and natural food preservative. Cumin also has been used for flavoring foods, salad, and dairy products in many countries (Johri, 2011). Accordingly, the *C. cyminum* EO (CcEO) and its major components can be used as safe natural compounds in the food industry as a replacement for synthetic preservatives and additives (Iacobellis et al., 2005). In addition, researches have shown that this plant has various pharmacological activities including excellent antioxidant (De et al., 2003; Megraj et al., 2011), antibacterial (Hajlaoui et al., 2010; Wanner et al., 2010), antifungal (Pai et al., 2010), and analgesic properties (De et al., 2003).

Accordingly, the first purpose of the present study was to determine the antimicrobial properties of the CcEO against *S. aureus* MDR clinical isolates and standard strains. To study the mechanism of antimicrobial properties of the CcEO, some virulence-related experiments were applied. For this aim, efflux pump inhibitory (EPI), QSI, and PIA inhibition activities of the CcEO were evaluated against tested organisms. Finally, major bioactive components of the CcEO were determined.

## Materials and Methods

### Ethics Approval

The studies involving human participants were reviewed and approved by the Ethics Committee for Research at Hamedan University of Medical Sciences with the ethical approval No IR.UMSHA.REC.1395.145. The next of kin of the patients/participants provided written informed consent to participate in this study.

### *C. cyminum* L. EO

*Cuminum cyminum* L. seeds were obtained from Shahid Beheshti Plant Institute, Tehran, Iran. To obtain the EO, the dehydrated powdered seeds of *C. cyminum* L. (100 g) were located in a distillation apparatus containing 1 L of distilled water and hydrodistilled using Clevenger-type apparatus for about 3 h according to the standard protocol (El Gendy et al., 2015). Afterward, the oil was gently removed and kept at 4°C in sterile dark glasses until being tested.

### Gas Chromatography–Mass Spectrometry Analysis

These EOs were analyzed by gas chromatography–mass spectrometry (GC-MS) analysis using a Hewlett Packard 5972A mass selective detector coupled with a Hewlett Packard 6890 gas chromatograph, equipped with a cross-linked 5% PH ME siloxane HP-5MS capillary column (30 m × 0.25 mm, film thickness 0.25 μm). The GC was done with the following conditions: carrier gas, helium with a flow rate of 2 mL/min; column temperature, 60, 275°C at 4°C/min; injector and detector temperatures, 280°C; volume injected, 0.1 μL of the oil; and split ratio, 1:25. The MS operating parameters were as follows: ionization potential, 70 ev; ion source temperature, 200°C; resolution, 1,000. Identification of components in the oil was based on GC retention indices relative to n-alkanes and computer matching with the Wiley 275.L library, as well as by comparison of the fragmentation patterns of the mass spectra with those reported in the literature (Kabouche et al., 2009).

### Bacterial Isolates and Culture Media

Ten *S. aureus* clinical and food-related isolates that had previously been isolated by our research group were included in the present study (Sharifi et al., 2018a). These strains were isolated from two sources over a period of 24 months from 2011 to 2013: (1) different clinical samples from patients admitted to the Hamedan hospitals, Iran; (2) mastitis bovine milk, Hamedan farms, Iran. For this study, MDR isolates were selected based on the Clinical and Laboratory Standards Institute definition for MDR strain (MDR defined as acquired non-susceptibility to at least one agent in three or more tested antimicrobial categories) (Magiorakos et al., 2012). Of 10 *S. aureus* isolates included in the present study, 8 (80%) were methicillin-resistant *S. aureus*. In addition, *S. aureus* ATCC 33591 (MDR strain) was used as positive control for all tests except for NorA EPI activity testing. *S. aureus* SA1199B (the fluoroquinolone-resistant NorA overexpressed strain) was used for NorA EPI activity assay. These standard strains were obtained from Persian Type Culture Collection, Tehran, Iran. All bacterial culture media were purchase from Merck, Germany.

### Determination of MIC and Minimum Bactericidal Concentration Values

The MICs for CcEO were determined by the broth microdilution method as described previously (Duarte et al., 2015). Briefly, 2-fold serial dilutions of CcEO (from 20 to 0.15 μL/mL) were prepared in 96-well plates by Müeller Hinton broth (MHB) and 2% of dimethyl sulfoxide (DMSO). Bacterial suspensions were prepared with a turbidity of 0.5 McFarland by diluting in MHB and added to each well to yield a final concentration of 4 × 10^5^ to 5 × 10^5^ colony-forming units per milliliter in wells. In addition, MHB-DMSO was used as growth controls. The plates were incubated at 37°C for 18–24 h under aerobic conditions. After the incubation period, the bacterial growth was visually assessed. The MIC was defined as the lowest concentration of CcEO without visible growth. In order to determine the minimum bactericidal concentration (MBC) from the wells without visible growth, 10 μL was plated on the tryptic soy agar media, and after incubation, the number of colonies was counted. The MBC was defined as the lowest compound concentration that caused the death of 99.9% of the bacterial inocula. The vancomycin (Sigma–Aldrich, St. Louis, MO, United States) was used as control for both MIC and MBC determination tests. These tests were repeated in three independent times.

### Scanning Electronic Microscopy

In this part of the study, the effect of CcEO on the structure and morphology of *S. aureus* ATCC 33591 cells at 1/2 MIC concentration was evaluated using scanning electron microscope (SEM) images. At first, the *S. aureus* cells were prepared in six-well plates, however, each well contained a glass coverslip this time. The control wells contained medium with bacteria (without CcEO), and the treated groups contained medium with 2.5 μg ⋅ mL^–1^ CcEO and bacteria. After 24 h of incubation at 37°C, the samples were removed and fixed in 2.5% buffered glutaraldehyde for 2.5 h followed by dehydration in graded ethanol. The samples were then dried at room temperature and glued onto stubs. At last, the processed samples were sputter-coated with gold and examined in a JEOL JSM-840 SEM operating at an accelerating voltage of 15 kV.

### EtBr Efflux Inhibition Assay

The test was performed according to Kaatz et al. (2000), with some modification. In details, *S. aureus* SA−1199B cells were grown overnight in MHB until an optical density at 600 nm of 0.6 was achieved, followed by centrifugation at 5,000 × *g* for 5 min. Then, pellets were suspended in 2 mL of phosphate-buffered saline (pH 7). Bacterial suspensions were vortexed and then transferred to 96-well plates followed by addition of a saline solution containing EtBr (8 μg ⋅ mL^–1^) and CcEO (2.5 μg ⋅ mL^–1^). Plates were placed in a Corbett Life Science Rotor-Gene 6000 Cycler (Qiagen, Germany) with excitation at 518 nm and emission at 605 nm. Differences in relative final fluorescence between CcEO-containing samples and control assays (samples containing EtBr alone and free of CcEO) were indicative of activity of CcEO to inhibit efflux of EtBr.

### Quantitative Real-Time Reverse Transcription–Polymerase Chain Reaction

The expression levels of hld, ica, and norA were examined in treatment with 1/2 MIC of the CcEO. For RNA isolation, the *S. aureus* cells were grown with and without the 1/2 MIC of CcEO in six-well polystyrene tissue culture plates containing broth medium with DMSO and incubated at 37°C for 24 h, and then the planktonic cells were removed and immediately processed for RNA extraction using a commercial RNA extraction and purification kit (SinaClon, Iran) according to the manufacturer’s instructions. Subsequently, the quantity and quality of the extracted RNA were approved by the agarose gel electrophoresis and also by measuring the absorbance at 260/280 nm using a Nanodrop spectrophotometer (ND-1000, Thermo Fisher Scientific, Waltham, MA, United States). The extracted RNAs were stored at −70°C until further analysis. After that, the purified RNAs were reverse transcribed to cDNA using a commercial cDNA synthesis kit according to the manufacturer’s instructions (Takara, Japan), and the obtained cDNA was stored at −70°C until used as DNA templates in the real-time reverse transcription–polymerase chain reaction (PCR) reactions.

The real-time PCR assay was performed using a commercial SYBR Green master mix (Amplicon, Denmark) and previously described pairs of primers (Table 1). The reactions were conducted in a Corbett Life Science Rotor-Gene 6000 Cycler (Qiagen), and for tested bacteria, the *16S rRNA* housekeeping gene was considered as an internal control to normalize the expression of target genes. The efficacy of the real-time PCR was calculated by the following formula: *E* = 10(−1^/slop)^ − 1. Then, the standard curves were optimized, and after that, the main reaction was performed. A negative control was also included in each run. Also, the specificity of the real-time PCR product was checked by gel electrophoresis, as well as the post-PCR melting-curve analysis under the following conditions: temperature starting at 60°C for 10 s followed by 0.5°C/10 s rising up to 95°C. All the samples were analyzed in triplicate, and finally, relative gene expression was calculated using the 2^–ΔΔCT^ method as described previously by Livak and Schmittgen (2001).

**TABLE 1:** Primers used for the quantitative real-time reverse transcription–polymerase chain reaction assay.

Genes	Sequence (5′–3′)	Annealing temperature, °C	References
*hld*	ATTTGTTCACTGTGTCGATAATCC	56	Kolar et al., 2013
	GGAGTGATTTCAATGGCACAAG		
*ica*	GGAAGTTCTGATAATACTGCTG	56	Harapanahalli et al., 2015
	GATGCTTGTTTGATTCCCTC		
*norA*	ATGAATAAACAGATTTTTGT	43	Truong-Bolduc et al., 2011
	TGATGTTATCGAGAGTGATT		
*16S rRNA*	AGCCGACCTGAGAGGGTGA	59	Koprivnjak et al., 2006
	TCTGGACCGTGTCTCAGTTCC		

### Statistical Analysis

Statistical analyses were performed using GraphPad Prism 6 software (GraphPad Software, San Diego, CA, United States). All experiments were performed in triplicate in at least two independent times. Analysis of variance was used to analyze the data, and *p* < 0.05 was considered to be statistically significant.

## Results

### Antibacterial Activity

**FIGURE 1:**
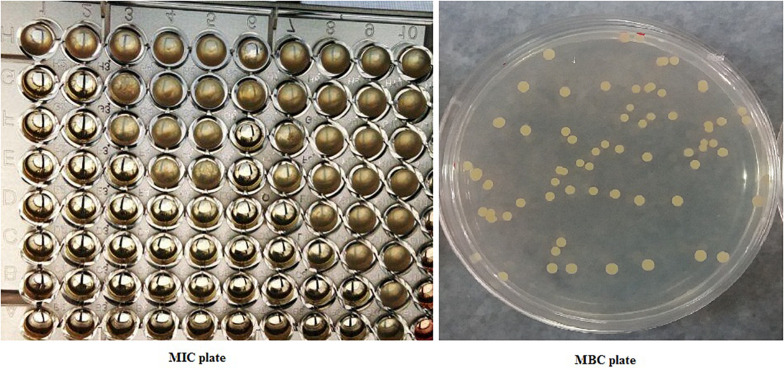
Plates for determining minimum inhibitory concentration and minimum bactericidal concentration.

*In vitro* bacteriostatic and bactericidal properties of the CcEO were evaluated against *S. aureus* bacteria (Figure 1). The CcEO exhibited excellent antibacterial properties, and the MICs and MBCs ranged from 1.25 to 5 and 2.5 to 5 μL ⋅ mL^–1^, respectively (Table 2).

**TABLE 2:** MIC and MBC values of the *Cuminum cyminum* L. essential oil (CcEO) against *S. aureus* bacteria.

Bacteria	CcEO		Vancomycin	
		
	MIC (μg ⋅ mL^–1^)	MBC (μg ⋅ mL^–1^)	MIC (μg ⋅ mL^–1^)	MBC (μg ⋅ mL^–1^)
Clinical isolates (*n* = 10)	1.25–5	2.5–5	1.25−2.5	1.25−5
*S. aureus* ATCC 33591	5	20	5	10
*S. aureus* SA1199B	5	20	5	10

*CcEO, *Cuminum cyminum* L. essential oil; MBC, minimum bactericidal concentration; MIC, minimum inhibitory concentration.*

### SEM Observation

**FIGURE 2:**
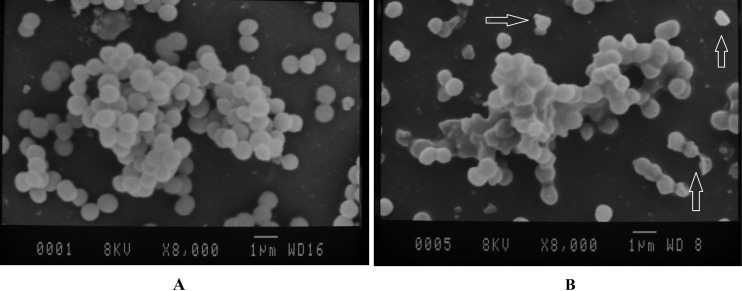
SEM images of *S. aureus* on the coverslips. **(A)** Negative control (untreated bacteria). **(B)** Treated with the 1/2 MIC concentration of the CcEO (magnification: ×8,000). MIC, minimum inhibitory concentration.

The surface morphology of *S. aur*eus cells was examined by the SEM analysis. The electron micrographs of both control and CcEO-treated microbial cells are shown in Figure 2. In the control groups, the untreated cells had the typical structure, showing a smooth wall for *S. aureus* (Figure 2A). In contrast, obvious harmful effects on the morphology of cell membranes were presented when strains were treated with the CcEO. Deformed, incomplete, and pitted shapes were observed in the treated cells (Figure 2B).

### NorA Efflux Pump Inhibitory Activity

Using EtBr efflux inhibition assay, the *S. aureus* NorA efflux pump was phenotypically affected by the CcEO treatment. The results showed that the CcEO significantly inhibited NorA efflux pump.

### Virulence Gene Inhibitory Activity

The effect of sub-MIC concentration of the CcEO on the expression of *hld*, *ica*, and *norA* genes was measured. Our results showed that 1/2 MIC concentration of the CcEO caused a significant downregulation of two of three (*hld* and *ica*) investigated genes (*p* < 0.05). Treatment of *S. aureus* with 0.625 to 1.25 μL ⋅ mL^–1^ of the CcEO resulted in −3.13- and −2.33-fold decreases in the expression of hld and ica, respectively. On the other hand, although norA expression decreased slightly under the effect of the CcEO, this downregulation was not statistically significant (−1.8-fold).

### Chemical Composition of the EO

In GC-MS analysis, 27 substances were detected. Based on our results cuminic aldehyde (38.26%), α,β-dihydroxyethylbenzene (29.16%), 2-caren-10-al (11.20%), γ-terpinene (6.49%), and β-pinene (5.25%) were the most detected compounds (Table 3).

**TABLE 3:** Chemical compositions, retention time, and percentage of the identified components from the CcEO.

Peak	Ret time	Name	%
1	6.214	Cumene	0.01
2	6.286	α-Thujene	0.07
3	6.394	Cyclohexane	0.02
4	6.517	α-Pinene	0.19
5	7.739	1-Phenylethanol	0.02
6	7.924	Sabinene	0.11
7	8.053	β-Pinene	5.25
8	8.602	β-Myrcene	0.23
9	9.105	Phellandrene	0.15
10	9.331	δ-3-Carene	0.01
11	9.614	α-Terpinene	0.03
12	9.814	*trans*-*p*-Menth-2-ene	0.01
13	9.989	Benzene, 1-methyl-4-(1-methylethyl)	4.33
14	10.127	β-Phellandrene	0.15
15	10.204	1,8-Cineole	0.08
16	11.478	γ-Terpinene	6.49
17	11.776	*trans*-Sabinene hydrate	0.04
18	12.7	α-Terpinene	0.03
19	13.136	Terpineol, Z-beta	0.08
20	16.731	4-Terpineol	0.33
21	17.378	p-Menth-1-en-8-ol	0.09
22	17.496	4-Isopropyl-1,3-cyclohexadien-1-yl	0.64
23	19.109	1,3-Benzenediol, 4-ethyl	0.38
24	20.233	Cuminic aldehyde	38.26
25	22.221	2-Caren-10-al	11.20
26	22.781	α,β-Dihydroxyethylbenzene	29.16
27	24.208	p-Mentha-1,4-dien-7-ol	0.16
Total			97.52

## Discussion

Several investigations have examined the antimicrobial effects of the CcEO (Gachkar et al., 2007; Derakhshan et al., 2010; El Gendy et al., 2015; Al-Snafi, 2016; Saee et al., 2016); nonetheless, there are a limited number of researches investigating the effect of this oil on the various virulence factors at sub-MIC concentrations. Thus, it is an open gate for more investigations. In the present study, the antibacterial, QS inhibitory, PIA production inhibitory properties, and NorA efflux pump inhibitory of the CcEO were investigated against MDR *S. aureus*. Also, the compositions of the CcEO were determined using GC-MS.

Antibacterial experiments demonstrated that the CcEO was effectual against tested *S. aureus* in which MIC and MBC were 1.25 to 5 μL ⋅ mL^–1^ and 2.5 to 20 μL ⋅ mL^–1^, respectively. In a study performed by Saee et al. (2016), the CcEO has admissible antibacterial activity against some uropathogenic isolates with MIC from 5 to 250 μL ⋅ mL^–1^. Another study reported that the CcEO and alcoholic extract of cumin seed have an antibacterial activity against planktonic and also biofilm forms of *Klebsiella pneumoniae* (Derakhshan et al., 2010). In addition, Gachkar et al. (2007), indicated that the EO extracted by hydrodistillation from Iranian *C. cyminum* displayed good antimicrobial activities against tested *Escherichia coli*, *S. aureus*, and *Listeria monocytogenes* (MIC = 1−2 μL ⋅ mL^–1^) (Gachkar et al., 2007). Along with the aforementioned reports, our results are in agreement with those of Singh et al. (2002), who reported that CcEO is equally good or more effective compared with standard antibiotics, at a low concentration. Based on GC-MS analysis, cuminic aldehyde (38.26%), α,β-dihydroxyethylbenzene (29.16%), 2-caren-10-al (11.20%), γ-terpinene (6.49%), and β-pinene (5.25%) were the most detected compounds. Studies on the antibacterial properties of cuminic aldehyde have shown that this compound can inhibit the growth and can cause death of Gram-positive and Gram-negative bacteria (Wanner et al., 2010). In addition, it has been shown that the antimicrobial activity of CcEO could be linked to the level of cuminic aldehyde (Wanner et al., 2010). Another detected substance, α-pinene, is an organic compound of the polyphenolic (terpene) group, and previous studies have shown the antibacterial properties of this compound (Leite et al., 2007). In addition, α-pinene also shows a significant activity in the modulation of antibiotic resistance by multiple mechanisms including inhibition of microbial efflux, decreased membrane integrity, and metabolic disruption (Pereira et al., 2017).

For the evaluation of biological activity, it is important to consider differences in the chemical composition of EOs extracted from plants of the same species. These differences arise from various factors including geographical situation, harvest time, ground conditions, genetic factors, and extraction methods (Sefidkon et al., 2007). In a study conducted by Rana (2014), the main compounds of CcEO from India were cuminaldehyde (49.4%), *p*-cymene (17.4%), β-pinene (6.3%), α-terpinen-7-al (6.8%), γ-terpinene (6.1%), p-cymen-7-ol (4.6%), and thymol (2.8%). In a similar study, α-pinene (29.1%), limonene (21.5%), 1,8-cineole (17.9%), and linalool (10.4%) were the main compounds of CcEO (Gachkar et al., 2007). Based on another study, the main compounds of the CcEO were cuminic aldehyde, γ-terpinene, β-pinene, *p*-cymene, and two *p*-menthadienol isomers (Wanner et al., 2010).

Antibacterial properties of the CcEO were also checked using SEM in treated *S. aureus* bacteria compared to controls. The results of this experiment showed that the CcEO at 1/2 MIC concentration (2.5 μg ⋅ mL^–1^) has a destructive effect on the bacterial cell wall structure and causes wall incoherence (Figure 1). This interference with the external structures of the bacteria may be due to the amphipathic nature of some CcEO compounds including alkaloid, flavonoid, and tannin (Al-Snafi, 2016). By these properties, the CcEO can penetrate to the hydrophobic region of the cytoplasmic membrane, which leads to pore formation or voltage-gated channels, resulting in the leakage of essential cellular components or passage of hydrophobic compounds across the cell membrane, and finally, cell death happens (Nazzaro et al., 2013; O’Bryan et al., 2015).

In the next part of the study, the QSI properties of the CcEO were investigated against *S. aureus*. To do this, the expression of QS functional gene (*hld*) was evaluated in treatment with 1/2 MIC of the EO versus untreated cells. Our results showed a significant downregulation of *hld* following treatment with 1/2 MIC concentration of the CcEO (−3.13-fold). As the QS system is different in Gram-positive and Gram-negative bacteria (different functional molecules and different receptors), the study of the QSI properties in these two groups of bacteria should be done separately (Haque et al., 2019). Interestingly, the QSI activity of this plant against Gram-negative bacteria was reported previously (Bouyahya et al., 2017). QSI potential of the South Indian common spice *C. cyminum* was reported against the QS-dependent phenotypic expressions in Gram-negative bacteria *Chromobacterium violaceum*, *Pseudomonas aeruginosa* PAO1, *Proteus mirabilis*, and *Serratia marcescens* (Bouyahya et al., 2017).

As, for the QSI activity, agents are used at sub-MIC concentration (1/2 MIC), it does not impose any selection pressure; therefore, resistance to these compounds does not occur (Sharifi et al., 2018a). With this background in mind, QSI has been a novel strategy to control various bacterial infections (Koh et al., 2013). According to importance of this matter, many efforts were done to find plant compounds with the QSI properties (Dorman and Deans, 2000). In this context, the QSI activity of some plant species, such as garlic, ginger, and turmeric, has been shown (Koh et al., 2013). Besides, the QS system plays a critical role in several virulence factors including bacterial toxin production and secretion, antibiotic resistance, and biofilm formation (Rutherford and Bassler, 2012); therefore, based on our results, the CcEO can be used for modulation of these factors of *S. aureus*.

Another tested virulence gene was *ica*, which codes PIA, the main substance in the structure of biofilms (Cramton et al., 1999). Our results showed significant decrease (−3.3-fold) in the expression of this gene, indicating that the CcEO could potentially inhibit the biofilm formation and biofilm development. In our previous studies in this field, the antibiofilm properties of the CcEO were obtained (unpublished data), which could be expected because of the QSI activity and also PIA production inhibitory activity potential. In fact, the antibiofilm properties of a substance can be achieved by different manners including inhibition of cell attachment (Jansen and Kohnen, 1995), inhibition of bacterial growth and proliferation (Roy et al., 2018), QSI activity (He et al., 2012), inhibition of exopolysaccharide production (Swamy et al., 2016), and use of matrix-degrading materials (Kaplan, 2010).

According to literature, the activity of efflux pumps is one of the most important factors in the development of antibiotic resistance in pathogens (Costa et al., 2016; Espinoza et al., 2019). It has been shown that NorA efflux pump, which belongs to the major facilitator superfamily, is a predominant efflux pump of *S. aureus* (Espinoza et al., 2019). In the present study, the inhibition of this pump in the NorA overexpressed strain SA1199B was evaluated by two phenotypic and genotypic methods.

In applied phenotypic test (EtBr efflux test), EtBr expelling in the CcEO-treated samples was significantly less than that in the control. On the other hand, quantitative PCR assay showed that the CcEO did not significantly affect the level of *norA* expression in treated strain compared to the control.

Therefore, the CcEO may induce conformational change in the NorA ef?ux pump structure, and as the accurate structure of the pump is necessary for efflux activity, any structural change will cause inactivity or reduced activity (Venter et al., 2015). Many studies on medicinal plant extracts and EOs showed the existence of accepted molecules that block efflux pumps in both Gram-positive and Gram-negative bacteria and possibly restore the antibiotic susceptibility, thus helping to treat infectious diseases by low or moderate concentration of antibiotics (Venter et al., 2015; Sharifi et al., 2016; Seukep et al., 2019). Because of the low number of studies related to the CcEO, it is difficult to draw a conclusion on the NorA EPI activity of this substance, warranting more investigations.

## Conclusion

With respect to the development of bacterial antibiotic resistance, the attempt for funding natural compounds from medicinal plants that possess antibacterial and antivirulence properties is a need. Accordingly, the present study investigated these mentioned properties of the CcEO. Based on the results, the CcEO not only had bacteriostatic and bactericidal properties, but also at sub-MIC concentrations, it blocked or suppressed different virulence factors in MDR strains of *S. aureus*. The affected mechanisms in the present study included QS system, PIA production, and NorA efflux pump, which are all influenced by the CcEO treatment. Taken together, after *in vivo* investigations to approve safety of the substance, CcEO might be considered as interesting sources of antibacterial and antivirulence components against MDR *S. aureus*.

## Data Availability Statement

The raw data supporting the conclusions of this article will be made available by the authors, without undue reservation.

## Author Contributions

AS performed the laboratory experiments and wrote the manuscript. AM designed, supervised the research, and edited the manuscript. TS and PM provided scientific consultations, and edited the manuscript. AN performed SEM examinations.

## Conflict of Interest

The authors declare that the research was conducted in the absence of any commercial or financial relationships that could be construed as a potential conflict of interest.

## Publisher’s Note

All claims expressed in this article are solely those of the authors and do not necessarily represent those of their affiliated organizations, or those of the publisher, the editors and the reviewers. Any product that may be evaluated in this article, or claim that may be made by its manufacturer, is not guaranteed or endorsed by the publisher.
